# Aligning public financial management system and free healthcare policies: lessons from a free maternal and child healthcare programme in Nigeria

**DOI:** 10.1186/s13561-019-0235-9

**Published:** 2019-06-13

**Authors:** Daniel Chukwuemeka Ogbuabor, Obinna Emmanuel Onwujekwe

**Affiliations:** 1 Department of Health Systems and Policy, Sustainable Impact Resource Agency, University of Nigeria Enugu Campus (UNEC), 22 Ogidi Street, Asata, Enugu, P.O. Box 15534, Enugu State, Nigeria; 2 Department of Health Administration and Management, University of Nigeria Enugu Campus, Enugu, Enugu State Nigeria; 3 Health Policy Research Group, College of Medicine, University of Nigeria Enugu Campus, Enugu, Enugu State Nigeria

**Keywords:** Public financial management, Free healthcare, Policy implementation, Nigeria

## Abstract

**Background:**

Relatively little is known about how public financial management (PFM) systems and health financing policies align in low- and middle-income countries. This study assessed the alignment of PFM systems with health financing functions in the free maternal and child healthcare programme (FMCHP) of Enugu State, Nigeria.

**Methods:**

Data were collected through quantitative and qualitative document review, and semi-structured, in-depth interview with 16 purposively selected policymakers involved in FMCHP. Data collection and analysis were by guided a framework for assessing alignment of PFM systems and health financing policies. Revenue and expenditure trend analyses were done using descriptive statistics and analysis of variance (ANOVA). Level of significance was set at ρ < 0.05. Qualitative data were analysed using a framework approach.

**Results:**

The results showed that no more than 50% of FMCHP fund were collected despite that the promised fund remained unchanged since inception. Revenue generation significantly varied between 2010 and 2016 (ρ < 0.05). Level of pooling was limited by non-compliance with contribution rules, recurrent unauthorised expenditure and absence of expenditure caps. The unauthorised expenditure significantly varied between 2010 and 2016 (ρ < 0.05). Misalignment of budget monitoring and purchasing revealed absence of auditing and delays in provider payment. Refunds to providers significantly varied between 2010 and 2016 (ρ < 0.05) due to weak Steering Committee, weak vetting team, paper-based claims management and institutional conflicts between Ministry of Health and district-level officials.

**Conclusions:**

This study identified important lessons to align PFM systems and FMCHP. A realistic and evidence-informed budget and enforcement of contribution rules are critical to adequate and sustainable revenue generation. Clarity of roles for various FMCHP committees and use of clear resource allocation strategy would strengthen pooling and fund management. Enforcement of provider payment standards, regular auditing, and a stronger role for the parliament in budgetary processes are warranted.

**Electronic supplementary material:**

The online version of this article (10.1186/s13561-019-0235-9) contains supplementary material, which is available to authorized users.

## Background

In 2007, Enugu State launched the free maternal and child healthcare programme (FMCHP) to improve financial protection and equity in the use of maternal and child health (MCH) services in publicly-owned health facilities that usually charges user fees [1]. Financial constraints significantly limited access to MCH services in Nigeria [2, 3]. Hence, free care policy implies that users do not pay for essential MCH services and drugs, when available, at point of service delivery. The programme is tax-funded through monthly state and local governments’ (SLG) contributions. The FMCHP is governed by a Steering Committee (SC), responsible for oversight, fund management and primary purchasing, and housed within the policy development and planning directorate (PDPD) of the Ministry of Health (MOH). A State Implementation Committee (SIC), housed within the State Health Board (SHB), monitors district-level implementation of FMCHP and serves as financial intermediary between the SC and service providers.

The flow of funds from FMCHP fund to providers has been fully described in a previous paper [4]. In a nutshell, healthcare providers are paid fees for each patient who received free services based on approved fee schedule. Healthcare providers duly record all transactions and submit monthly claims to the SIC for vetting. Vetted claims are approved by the SC. The SC transfers funds to the SIC to pay providers their approved claims. Whereas healthcare providers receive 70% of the cost of services, the balance of 30% is used to defray administrative costs. The FMCHP funds, as all public funds, is subject to Enugu state’s public financial management systems and rules including budgeting, financial instructions, financial reporting and auditing.

Public financial management (PFM), described as institutions, policies and processes governing the use of public funds, influence how health financing policies contribute to universal health coverage (UHC) [5–8]. Countries that has made significant progress towards UHC relied on a dominant share of public funds to finance health [9]. Functional PFM systems would ensure that funds meant for health financing policies are adequate and predictable, equitably and efficiently used, better accounted for [5–8]. In contrast, when PFM systems and health financing policies are misaligned, UHC schemes may not be prioritized in the budget, resource allocation might be unpredictable and fund management ineffective and inefficient. Such weak PFM systems may result in ineffective implementation of health financing policies in support of UHC due to significant resource leakages and misuse of public funds [5–8].

Evidence of (mis) alignment of PFM and health financing policies in low and middle-income countries are growing. Funding for UHC schemes in China, Thailand and Eastern European Countries increased and were predictable [10–12], which contrasts evidence of insufficient budgetary allocations and underfunding from Ghana, Nicaragua and India [13–15] and unchanging annual government spending on free care policy in Senegal [16]. State governments defaulted from payment of their contributions to UHC schemes in Nigeria and Mexico [17–19]. Whereas fixed annual budget and cap on provider payment controlled costs Thailand’s universal coverage scheme (UCS) [20], non-adherence to spending caps in Mexico’s Seguro Popular (SP) resulted in use of funds for unauthorised purposes, high public spending on drugs and contracting unauthorised personnel [19, 21]. Nevertheless, misuse of funds in Mexico’s SP necessitated the Ministry of Finance to keep resources out of local treasuries and instead, pay providers directly from resources in federal treasury [21].

The experiences of Mexico confirm that institutional conflicts in fund management, significant delays in transfer of funds from state to healthcare providers, limited financial information disclosure and high administrative cost result from misalignment of PFM systems and health purchasing [17, 19, 21]. Conversely, a low administrative cost was found in Thailand’s UCS because the scheme has no revenue-raising responsibility and underinvests in administrative functions [22]. In Vietnam, budgets based on historical expenditure of the preceding year resulted in lower fund allocation to providers than their actual healthcare expenditure [23]. In Thailand, hospital directors misallocated resources meant for contracted units of primary care [24]. Lack of administrative and service utilisation data constrained monitoring of free healthcare policies in India and Nigeria [15, 18], which contrasts experiences of robust health management information system in Thailand’s UCS [20].

The FMCHP policy envisaged that adherence to the public budgeting processes, contribution rules and state financial instructions would ensure predictable SLG budget transfers, transparent financial management and optimal use of FMCHP funds. However, evidence of inadequate funding of the FMCHP and weak commitment of Local Government Councils; declining number of health facilities reimbursed for free MCH services; and users continuing to pay for notionally free MCH services indicate that PFM systems and health financing functions in FMCHP are misaligned in Enugu State [25–27]. This paper explores these misalignments and provides evidence of how PFM can be better aligned with FMCHP objectives. Such insights can be used by health policymakers, public budget officials, health providers and development partners to ensure efficient and effective use of public funds to finance free healthcare policies in Nigeria and similar low-resource settings.

## Methods

### Conceptual framework

**Fig. 1:**
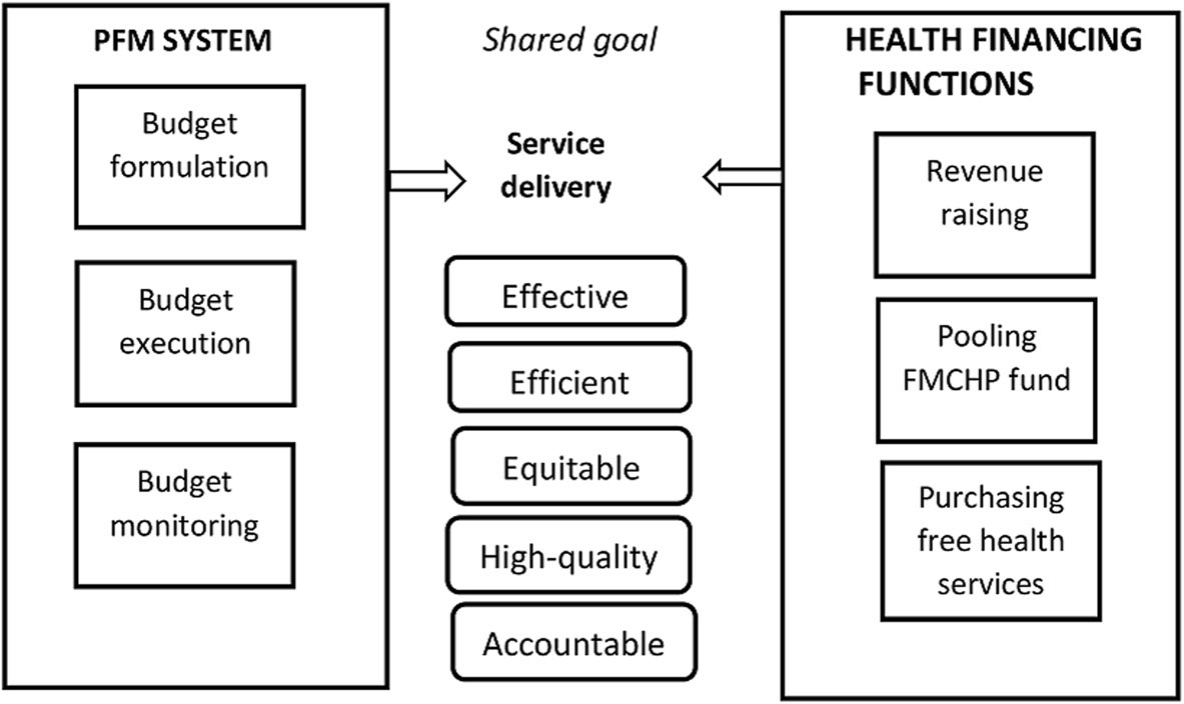
Framework for assessing alignment of public financial management and health financing policies

The study was guided by Cashin and colleagues’ framework for assessing alignment of public financial management (PFM) and health financing policies [28]. The framework integrates health financing functions and health sector financial management into the budget cycle (Fig. 1). The budget cycle has three stages: budget formulation, budget execution and budget monitoring. Budget formulation aligns with revenue raising in health financing and involves determining resource allocation to FMCHP. Budget execution aligns with pooling of FMCHP funds and involves transfer of approved funds to, and fund management at the MOH. Budget monitoring involves making payments to healthcare providers for free services delivered (purchasing) and ensuring compliance with purchasing rules and accountability of purchasing agencies. This framework was deemed appropriate because it addresses the specific PFM requirements of the health sector and provides functional approach for investigating how PFM and FMCHP could be better aligned to contribute to universal health coverage [28].

### Study setting

The study was conducted in Enugu State, Southeast Nigeria. Enugu State consists of seventeen (17) Local Government Areas (LGAs). The LGAs are delineated into 7 health districts and 68 local health authorities. Between 2008 and 2016, Enugu State population increased by about 28.7% at 3.2% growth rate of 2006 census estimate [29]. Children under 5 years and women of childbearing age (WCBA) constitute about 17% and 45% of the population respectively [30]. From 2008 to 2013, the proportion of currently pregnant women rose from 7.6% to 8.4%; the total fertility rate increased from 4.4 to 4.8; the proportion of women who are delivered in public health facility increased from 21.4% to 36.5%; and under-five mortality rate declined from 153 per 1000 livebirths to 131 per 1000 livebirths [30]. Publicly owned health facilities in each district health include a district hospital, cottage hospitals and primary health facilities. There is also a state teaching hospital in the capital city of Enugu.

### Research design

The study adopted a mixed methods design. The quantitative component consists of secondary analysis of financial and administrative data. The qualitative component included document review and in-depth, semi-structured interviews. Mixed methods was used because it would sufficiently capture the complexity of implementation processes and the findings could be triangulated [31].

### Study population and sampling strategy

The target population for in-depth interviews were policymakers involved in FMCHP implementation at the state and district levels. We purposively selected state-level policymakers (*n* = 12) from the SC and SIC of the FMCHP and district-level policymakers (*n* = 4) because of their position, involvement in administration of FMCHP and willingness to participate in the study. To facilitate selection of district-level policymakers, we divided the seven health districts into two contrasting clusters of well-performing and less-well-performing districts using provider payment data and randomly selected one district from each cluster [4, 25]. Two policymakers were selected from each of the two districts.

### Data collection

Quantitative data were abstracted from administrative and financial records collected from the MOH, SHB, Enugu State Teaching Hospital and State budget using an abstraction form. The abstraction form included data on SLG budget transfers to FMCHP fund, transfer from the FMCHP fund to SHB’s FMCHP account, other expenditure made from the FMCHP pool, payments to health facilities and central medical stores from SIC, vetted provider claims and audit report.

Qualitative data were extracted from policy documents, programme reports, Hansard and memoranda on FMCHP collected from the MOH and Enugu State House of Assembly. About 27 documents, purposively selected because they informed the research questions of this study, were reviewed (Additional file 1). The documents were identified in consultation with key MOH officials and clerk of the House Committee on Health**.**

We interviewed 16 policymakers using in-depth, semi-structured interview guide as a part of large assessment of governance of the FMCHP [25]. The interview guide included questions for assessing FMCHP budget formulation, release of funds to MOH, flow of funds from FMCHP fund to healthcare providers and monitoring of financial management rules. The participants were identified using government officials as gatekeepers. Interviews held in their offices, were conducted in English and lasted about one and half hours. The interviews were audiotaped, transcribed verbatim and the transcripts sent back to participants for validation.

### Data analysis

#### Quantitative component

We conducted financial trend analysis of revenue collection, pooling and purchasing. Descriptive statistics used included percentages and graphs. Analysis of variance (ANOVA) was used to measure statistical significance of mean differences in proportion of variables (population of target beneficiaries, revenue raised, pool size, unauthorised expenditure, paid claims and unpaid claims) at ρ < 0.05. Unauthorised expenditure in this study means spending from the FMCHP funds that are beyond the scope of FMCHP guidelines. Data were analysed using Statistical Package for Social Sciences (SPSS) version 20 (IBM, New York, USA).

#### Qualitative component

The interview data were imported into NVivo software (version 11, QSR International Pty Ltd., Victoria, Australia) and analysed using a framework approach [32]. Deductive and inductive coding strategies were used by two independent coders to fit data into categories and inconsistencies resolved by consensus. The main themes were deduced from the conceptual framework of the study. Inductive codes reflected sources of misalignments between PFM and health financing functions and were generated by reading the transcripts and assigning codes to emergent themes.

### Ethical consideration

The study was approved by the Health Research Ethics Committee of the University of Nigeria Teaching Hospital Enugu, Nigeria. Written, informed consent was obtained from all participants for both participation and audio-recording of interviews.

## Results

### Quantitative component

#### Budget formulation and revenue raising for FMCHP

**Fig. 2:**
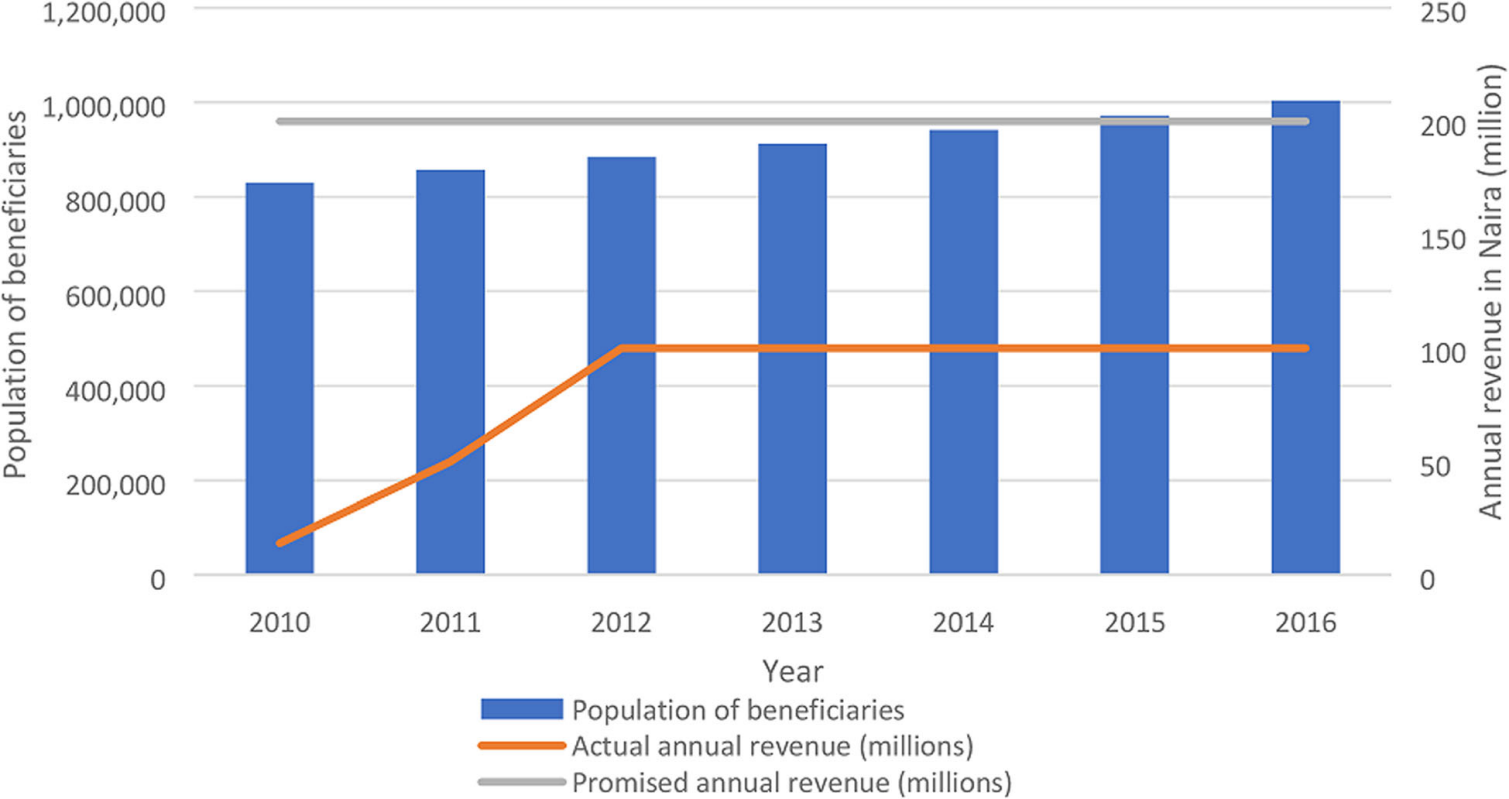
Trend of revenue raising for FMCHP and population of target beneficiaries

FMCHP revenue generation significantly varied between 2010 and 2016 (ρ < 0.05). Figure 2 shows that no more than 50% of the promised revenue (200 million naira per annum) were generated (averaging 41.29%) per annum, whereas the population of target beneficiaries significantly increased (ρ < 0.05) between 2010 and 2016.

#### Budget execution and pooling of FMCHP fund

**Fig. 3:**
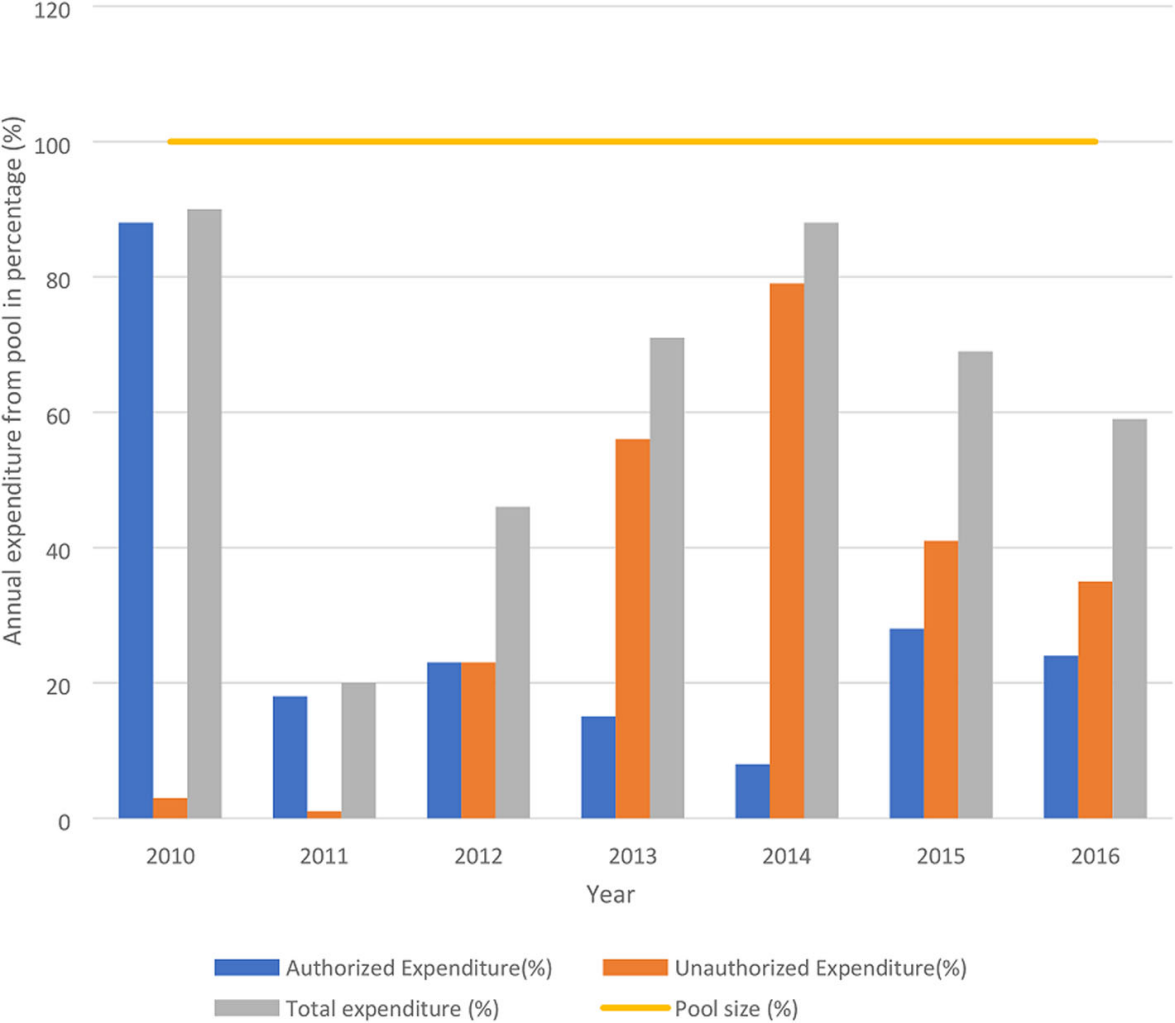
Trend of spending from FMCHP funds between 2010 and 2016

An average of 63% of annual pool size was spent between 2010 and 2016 ranging from 20% to 90%. The proportion of annual unauthorized expenditure significantly rose from 1% in 2011 to 79% in 2014 and declined to 35% in 2016 (ρ < 0.05) but remained higher than authorized expenditures between 2013 and 2016 (Fig. 3). The average unauthorized expenditure was 34% per annum.

#### Budget monitoring and purchasing in FMCHP

**Fig. 4:**
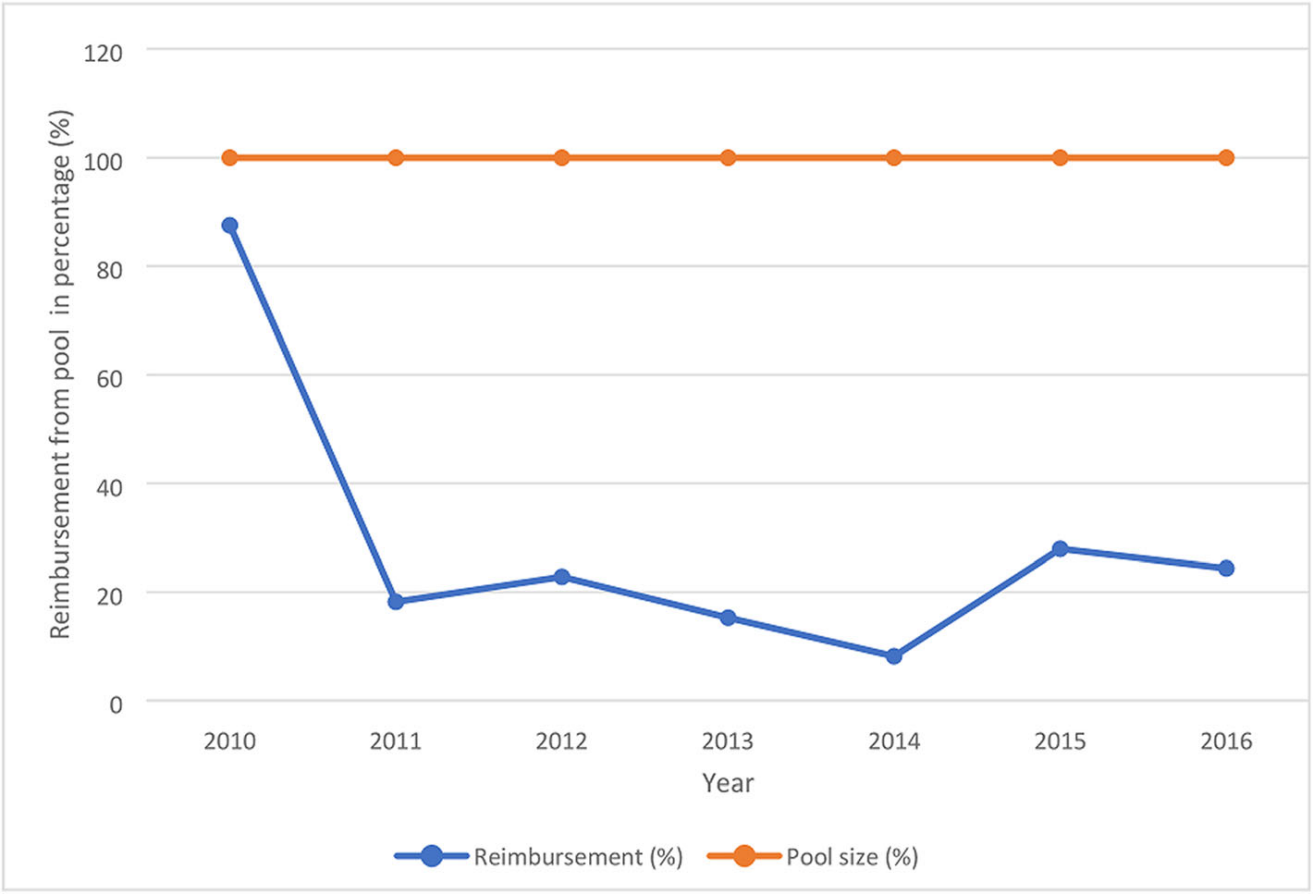
Proportion of annual pool size spent on payment of healthcare providers

**Fig. 5:**
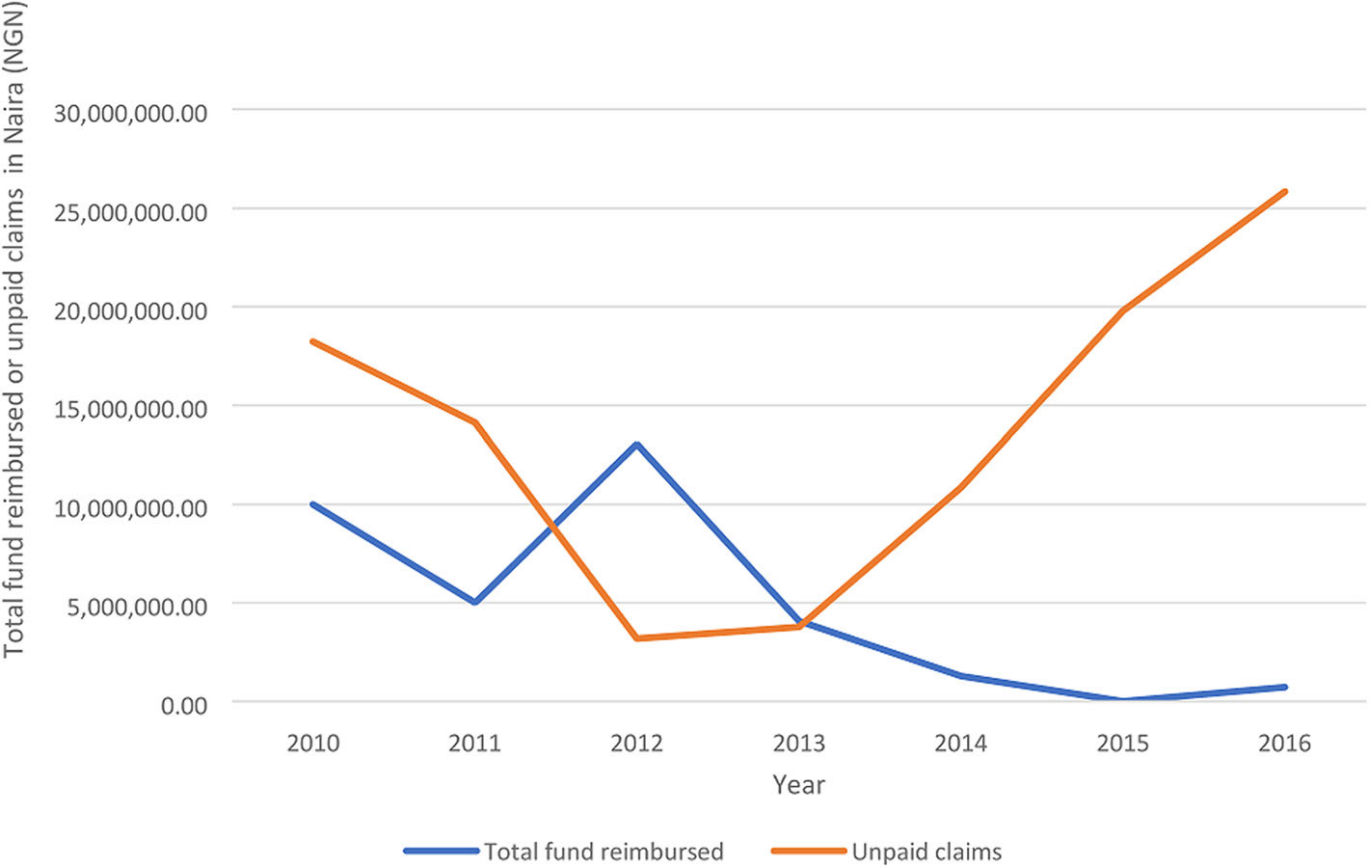
Trend of annual reimbursement and cumulative unpaid claims in ESUTH

The proportion of the annual pool size used to pay providers significantly varied between 2010 and 2016 (ρ < 0.05) (Fig. 4). Of the 17 reimbursement exercises, about 44% took a gap of 1 to 3 months, 31% took 4 to 6 months and 25% between 7 and 15 months. Most reimbursements included several unpaid claims for the preceding 2 to 3 years. Refunds to the state teaching hospital significantly declined from 2010 to 2016 (ρ < 0.05) and from 2011, is inversely related to unpaid claims (Fig. 5). The unpaid claims significantly increased from 2012 to 2016 (ρ < 0.05).

The administrative cost significantly rose from about 4% in 2013 to about 19% in 2016 (ρ < 0.05). Drug costs constitute bulk of FMCHP expenses but significantly declined from about 86% in 2013 to about 38% in 2016 (ρ < 0.05). The cost of services significantly increased from about 10% in 2013 to about 43% in 2016 (ρ < 0.05).

#### Qualitative component

Table 1 shows the key themes and sub-themes that characterise the misalignment of PFM system and health financing functions in FMCHP.

**Table 1:** Misalignment of PFM and health financing functions in FMCHP in Enugu State

PFM system	Health financing functions	Themes	Sub-themes
Budget formulation	Revenue raising	Level of funding	Weak budgeting with promised funding remaining static since inception
			Weak enforcement of revised contribution rule
Budget execution	Pooling and fund management	Level of pooling	Only Local Government sustained contribution to FMCHP fund
		Level of administrative efficiency	Weak Steering Committee
			No spending cap in the FMCHP guidelines
			High unauthorised expenditure from FMCHP fund
Budget monitoring	Purchasing	Payment of providers	Delayed payment of providers
			Fraction of claims paid to some providers
		Level of administrative efficiency	Non-remittance of administrative costs to LHAs
			Over-reporting of attendance by providers (gaming)
		Transparency	Unclear reimbursement process
			Lack of financial information disclosure
			No regular auditing of FMCHP account
			Resistance to financial monitoring committee from SIC officials

#### Budget formulation and revenue raising for FMCHP

Document review (DR) showed that FMCHP funding remained at 2008 cost estimate (DR3); the revenue raised were unpredictable, less than the promised fund and always in arears (DR3, DR4, DR6, DR15). In 2009, State Economic Planning Commission (SEPC) set new rules for direct deduction of SLGs’ contribution by Joint Accounts and Allocation Committee (DR14, DR15). That notwithstanding, only Local Governments’ contributions were deducted and transferred to FMCHP fund (DR14, DR15).

Most policymakers stated that annual budget for FMCHP were historical. Yet compliance with the contribution rule was weak; and the parliament lacked power to alter the budget estimates. Since 2010, only LGs contributed to the FMCHP fund, while “*the state government has not actually leaved up to its own responsibilities of making regular contributions”* (policymaker 3). Few policymakers indicated that funding ceiling remained unchanged since inception. As one policymaker observed, *“Free MCH budget should be reviewed every financial year – either upwards or downwards – but it had remained the same all through which does not look scientific or realistic”* (policymaker 10).

#### Budget execution and pooling of FMCHP fund

Review of documents indicated that rules for spending FMCHP funds covered services, drugs, laboratory services, vetting of facility claims and mobilisation and advocacy activities (DR1, DR5). Nonetheless, there are no spending caps for resource allocation in the guidelines. FMCHP funds were used to procure 11 vehicles in 2014 (DR14) and fund some activities of the MOH beyond the scope of FMCHP (DR 22). Yet, we found only one FMCHP audit report (DR22).

Most policymakers said that procedures for spending FMCHP fund are not adhered to. They reported that “*Steering Committee met only twice”* (policymaker 1) in 7 years and approvals of disbursement from FMCHP fund were done by Commissioner responsible for health. *“There were commissioners who delayed approval of reimbursement of providers even when there were lots of funds in the FMCHP account”* (policymaker 6). Yet, FMCHP funds were used to finance other health activities which are not authorized by FMCHP guidelines. *“When we received certain approvals from the State Governor without cash-backing, we normally took money from the FMCHP fund to finance them”* (policymaker 4)*.* The parliamentary committee on health monitors ministerial spending from FMCHP fund, but its role was limited to exposing inefficiencies.

#### Budget monitoring and purchasing in FMCHP

Review of documents indicated that at the inception of FMCHP, the SIC paid health facilities through their local health authorities (LHAs), but since 2010, providers were paid directly due to leakages at the LHAs (DR14, DR15). Funds accruing to LHAs to defray administrative cost were not remitted between 2010 and 2015 (DR23). Payment of providers are often late or never done; and most providers are unclear about the claim process (DR3). Some facilities are paid fractions of their claims (DR2). Payment uncertainties resulted in stock-outs of commodities and resumption of user fees in some facilities (DR2, DR10, DR14, DR17, DR19).

Most policymakers identified weak organizational capacity of the SC as obstacle to effective health purchasing. They indicated that SC rarely met, which constrained timely and predictable payment of providers. Approvals for payment of providers were done by the Commissioner responsible for health. Consequently, reimbursement *“timelines stipulated in the free care programme guidelines were not met and took more than six months after vetting”* (policymaker 2).

Most state-level policymakers explained that there were leakages in funds when LHA secretaries served as financial intermediaries for paying providers: *“we discovered that the LHA secretaries were keeping back part of the moneys. So that is why all the facilities were directed to open account”* (policymaker 1). District-level policymakers observed that *“since state-level policymakers by-passed LHA Secretaries in the reimbursement process, LHA Secretaries became aloof”* (policymaker 14) to provider accounting and financial reporting requirements.

Most policymakers observed that reimbursement processes are paper-based and not integrated into state health management information system. Claim forms that were not properly completed were kept aside while figures on mutilated pages of claims form were deducted from total claims before recommending vetted claims to SC for payment. Few policymakers observed that vetting team conducted quality assurance visits to “*verify that expenditure claimed in the reimbursement forms corresponded with facility records”* (policymaker 8). Sometimes, service data were inconsistent with providers’ claims, which is described as data “konjaring”, that is over-reporting attendance to increase claims (policymaker 10).

Most policymakers revealed that FMCHP financial information were not publicly disclosed and that the SIC was limited to *“writing and issuance of approved reimbursement cheques to health facilities”* (policymaker 10). Policymakers also stated that paying providers directly for service charges and through the central medical store for drugs, and existence of financial monitoring committee (FMC) enabled the SIC to comply with purchasing rules. Few policymakers observed that establishment of FMC resulted in conflictual relationship between the PDPD and SHB. *“The financial monitoring committee instructed that the Board should never issue cheque to any facility without reporting to the committee. The Board disregarded the directive”* (policymaker 8).

## Discussion

The findings showed that financing of FMCHP was insufficient and unpredictable. Consistent with experiences in Senegal [16], the level of promised funds remained unchanged since inception despite increases in population of target beneficiaries and changes in the unit cost of services and drugs due to rising inflation rates [33]. Changes in target population and increase in inflation rates imply underfunding of the scheme even if government transferred fully the existing budget commitment to the programme. Inflation rate affects fiscal policy behaviour in Nigeria [33], and may explain the weak compliance with contribution rules by SLG. Besides evidence of state governments’ defaulting in their contributions to UHC schemes in Nigeria and Mexico [17–19], other studies also found poor government commitment to funding UHC schemes consistent with findings of this study [15, 19]. Conversely, Thailand’s UCS budget increased substantially between 2002 and 2011 and is timely transferred to the scheme [10, 34]. In comparing Thailand’s UCS to Nigeria’s FMCHP, increased funding of UCS was due to increased annual fiscal capacities and evidence-informed negotiation of higher capitation rates [22]. Aligning PFM systems and revenue raising to support FMCHP would entail a shift from historical budgeting to formulating a realistic and evidence-informed annual budget for FMCHP, stronger role for the parliament and citizens, and strengthening enforcement of the contribution rules to guarantee appropriate and timely state budget transfer.

The study revealed that absence of clear resource allocation strategy, high unauthorised expenditure from the pool, and weak accountability between SC and SIC constrained efficient pooling and fund management. Lack of spending caps in FMCHP contrasts experiences in Mexico’s Seguro Popular, where resource allocation rule stipulates spending caps for human resources, pharmaceuticals and preventive activities [19, 21]. However, experiences in Mexico indicate that resource allocation rules would not necessarily translate to adherence to negotiated expenditure targets as implementers incurred huge unauthorised expenses [17, 19]. Similarly, this study confirms the Mexico’s experiences of use of funds for free care policy for unauthorised activities. The balance of power within the SC seem to have favoured the MOH to usurp the pooling and fund management function of the SC but resulted in huge unauthorised expenses from FMCHP funds and limited financial information disclosure. In addition, weak accounting and financial reporting from the SIC resulted in institutional conflict between the MOH and the SHB. The MOH set the FMC to ensure administrative efficiency in fund management and strengthen the logical link between pooling and purchasing. To better align PFM and pooling and fund management, there is a need for clarity of roles and responsibilities for various FMCHP committees, disclosure of financial information to various stakeholders, clear resource allocation strategy and stronger parliamentary oversight of FMCHP fund.

The study further revealed that misalignment of PFM systems and purchasing is characterised by weak budget evaluation and delay in reimbursing providers for free services. Although financial monitoring committee existed, its activities merely focused on compliance with financial procedures but not how equitably or efficiently funds are used. Similarly, the oversight role of parliamentary committee on health was limited to exposing non-compliance with PFM rules. It is imperative to audit FMCHP fund by an independent agency and examine how effectively and efficiently FMCHP funds have been used to achieve its policy objectives [8].

Provider payment delays arise from delay in accounting and financial reporting by providers, delay in vetting of provider claims and delay in approving and transfer of approved claims to providers. Similar delays in transfer of funds from the state to healthcare providers was found in Mexico [17]. Four factors seem to be influencing the delay in provider payment in this study. The first factor is institutional conflict between LHA secretaries and the MOH. At inception, LHA Secretaries served as financial intermediary between the SIC and providers and had substantial discretion in financial resource allocation to service providers. After 5 years of implementation (in 2012), the MOH commenced transfer of service charges directly to service providers due to allegations of misappropriation of funds by LHA secretaries similar to misallocation of resources to contracted units of primary care by hospital directors in Thailand’s UCS [24]. Consequently, the LHA Secretaries lost interest in monitoring and supervising the accounting and financial reporting by providers.

The second factor was weak vetting team. Delay in vetting of claims resulted from an initial lack of budgetary support for vetting team, incessant transfer of vetting team members, absence of incentives for the vetting team, weak quality assurance system, weak information and communication technology (ICT) support, and centralization of vetting of claims. Since the revised FMCHP policy in 2013 has provided for use of FMCHP funds to cover administrative costs of vetting claims, a meaningful change would be decentralization of vetting to health districts and linking district vetting offices to central coordinating vetting unit at State Health Board using functional ICT infrastructure.

The third factor is that the FMCHP claims’ management is paper-based process and has not been integrated into health management information system (HMIS). This study’s finding contrasts experiences in Thailand where evidence of utilization informs the capitation rates (20), but similar to experiences in India and Nigeria where lack of administrative and service utilization data constrained monitoring of free healthcare policies [15, 18]. Limited ICT infrastructure constrained accounting and financial reporting by providers, vetting of claims and transfer of funds to providers. Although HMIS is not an intrinsic part of provider payment system, it shapes the claims reporting and billing system [35].

The fourth factor is weakness of the SC. Approval of vetted claims is assigned to SC but in practice, Commissioner responsible for health approves disbursements from FMCHP fund. Thus, weak organizational capacity of SC constrained effectiveness and efficiency of purchasing because approvals depended on (un) willingness of the commissioner to approve funds. As we have argued elsewhere [4], making the purchasing agency an autonomous entity, consistent enforcement of provider payment standards and use of ICT aligned with HMIS to manage provider payment would realign PFM systems and purchasing objectives of the FMCHP.

The study has explored the misalignments between PFM systems and free healthcare policies through a detailed analysis the free maternal and child healthcare policy of Enugu state, south-east, Nigeria. The study has generated useful insights about how public budgeting rules, processes and practices influence free healthcare policies in resource-constrained settings, and the triangulation of quantitative and qualitative findings increases the validity of our conclusion that PFM plays key roles in the effectiveness of free healthcare policies. Evidence from this study may be limited by poor availability and accessibility of financial and administrative records of FMCHP. As an example, outstanding claims of district providers could not be analysed due to lack of data. However, the study leveraged on the first authors’ insider-researcher position to obtain timely, the financial records that informed the data reported in this paper.

## Conclusion

This study identified important lessons to align public financial management systems and free healthcare policies in Nigeria and similar settings. A shift from historical budgeting to a realistic and evidence-informed budget and enforcement of contribution rules would ensure sufficient and sustainable revenue generation for FMCHP. Clarity of roles and responsibilities for various FMCHP committees, disclosure of financial information to the various stakeholders, use of clear resource allocation strategy and adherence to fund management rules would strengthen pooling and fund management. Balancing revenue and expenditure, regular auditing, enforcement of provider payment standards and use of ICT aligned with HMIS to manage provider payment would guarantee timely payment of providers. A stronger role for the parliament in budgetary processes in FMCHP is warranted.

## Additional file

Additional file 1: List of documents reviewed (DOCX 14 kb)

## Data Availability

The datasets generated and/or analysed during the current study are not publicly available but are available from the corresponding author on reasonable request.

## Abbreviations

DR: Document Review
FMCHP: Free maternal and child healthcare programme
LG: Local Government
LGA: Local Government Areas
LHA: Local Health Authority
LHAs: Local Health Authorities
MCH: Maternal and child health
MOH: Ministry of health
PDPD: Policy Development and Planning Directorate
PFM: Public financial management
PHC: Primary health care
REPSS: State Health Protection Regime
SC: Steering Committee
SEPC: State Economic Planning Commission
SHB: State Health Board
SIC: State Implementation Committee
SLG: State and Local Governments
SP: Seguro Popular
UCS: Universal coverage schemes
UHC: Universal health coverage
WCBA: Women of childbearing age
