# Supplementary material for: Aligning public financial management system and free healthcare policies: lessons from a free maternal and child healthcare programme in Nigeria
Source: Health Econ Rev. 2019 Jun 13;9:17. doi: 10.1186/s13561-019-0235-9 (PMC6734425; doi:10.1186/s13561-019-0235-9)
Supplement: Supplementary file 1 — List of documents reviewed (DOCX 14 kb) [file 13561_2019_235_MOESM1_ESM.docx]

**List of policy documents reviewed**

1. Enugu state 2013 guidelines for implementation of free maternal and u-5 child health programme. (DR1).
2. PATHS2 consultancy report on revision of FMCH policy and implementation guidelines in Enugu State. (DR2).
3. Enugu state 2013 policy on free maternal and child health services in Enugu State. (DR3).
4. Enugu State free maternal and child healthcare program implementation committee report, 2008. (DR4).
5. Enugu state 2008 guidelines for implementation of free maternal and u-5 child health programme. (DR5).
6. Enugu state 2007 policy brief on free maternal and child health services in Enugu State. (DR6).
7. Enugu State free maternal and child healthcare program implementation update, 2010. (DR7).
8. Development of facility health committees to strengthen voice and accountability in Enugu State. PATHS2 Strategy Report. (DR8)
9. Facility health committees in Enugu state: operational guidelines. (DR9)
10. PATHS2 report of rapid assessment of 76 health facilities in six local government areas for implementation of essential systems and service packages. (DR10).
11. SMOH. (2008, November). Report of committee on engaging the private sector health care providers in the free maternal and child health programme (FMCH) of Enugu State. (DR11).
12. SMOH. (2009, May). Implementation guidelines for private sector participation in Enugu state free MCH programme. (DR12).
13. Annual performance review of Enugu state health sector. (DR13).
14. Implementation of the Enugu State free maternal and child healthcare program: the concept, operations, challenges and prospects. Memo by former permanent secretary SMOH to Enugu State House Committee on Health (DR14).
15. Memo on FMCHP by Executive Secretary, Enugu State Planning Commission to Enugu State House Committee on Health (DR15).
16. Memo on FMCHP implementation by Facility Health Committees’ Alliance to Enugu State House Committee on Health (DR16).
17. FMCHP: Prospects, challenges and recommendations. Memo by the Chief Executive Officers of District Health Boards to Enugu State House Committee on Health (DR17).
18. Enugu State free maternal and child healthcare programme: overview, considerations and recommendations for improvement. Memo by State Health Board to Enugu State House Committee on Health (DR18).
19. An in-depth enquiry into state and local government contribution to FMCHP fund management and re-imbursement of health facilities. Memo by Local Government Service Commission to Enugu State House Committee on Health (DR19).
20. Memo on FMCHP implementation by Health Reform Foundation of Nigeria (HERFON) to Enugu State House Committee on Health. (DR20).
21. ENHA. (2015, October). Hansard on motion no.HM/03/15 “immediate abolition of provision of evidence of tax payment as condition precedent for benefiting from Enugu state FMCHP in all state-owned health facilities. Enugu, Nigeria: Enugu State House of Assembly. (DR21)
22. Audit report on statement of account of FMCH with the State Health Board and FMCH account in the Ministry of Health, Enugu. (DR22).
23. Statement of account of FMCHP January 2010 to December 2016 from ESUT Teaching Hospital Parklane, Enugu. (DR23).
24. Statement of account of FMCHP January 2010 to December 2016 from State Ministry of Health, Enugu. (DR24).
25. Statement of account of FMCHP January 2010 to December 2016 from State Health Board, Enugu. (DR25).
26. Some FMCH vetted claims between 2010 to 2016 (DR26)
27. Drug revolving fund reports between 2013 and 2016 (DR27).
